# Aggressive Angiomyxoma of the Posterior Wall of the Uterus

**DOI:** 10.7759/cureus.12023

**Published:** 2020-12-11

**Authors:** Amal M Alosaimi, Hatim M Al-Jifree, Suzan Y Alharbi, Amirah S Algethami

**Affiliations:** 1 Department of Oncology, College of Medicine, King Saud Bin Abdulaziz University for Health Sciences, Jeddah, SAU; 2 Department of Oncology, King Abdullah International Medical Research Center, Jeddah, SAU; 3 ‏Department of Gynecologic Oncology, King Saud Bin Abdulaziz University for Health Sciences, Jeddah, SAU; 4 Department of Oncology, Ministry of National Guard Health Affairs, Jeddah, SAU

**Keywords:** angiomyxoma, mesenchymal tumor, stromal tumor

## Abstract

Aggressive angiomyxoma (AA) is a rare benign mesenchymal tumor that usually arises in the vulvovaginal and perineal regions of premenopausal females. The treatment of choice is surgical excision. Hormonal therapy or radiotherapy have emerged as alternative forms of treatment but are indefinite. In this article, we report a case of aggressive angiomyxoma in the posterior wall of the uterus of a 35-year-old Saudi female patient. The clinical data, imaging, histopathology, treatment, and prognosis were analyzed, and related literatures were reviewed. The frequency of recurrence in these tumors emphasizes the importance of long-term follow-ups.

## Introduction

Aggressive angiomyxoma (AA) is a rare benign slow-growing stromal tumor that usually arises in the female vulvovaginal and perineal region [1]. To the best of our knowledge, the number of cases reported in the literature is less than 350 [2]. Most of the AA cases reported in female patients involved the vulvovaginal region, perineum, and pelvis of women. In a review of 106 cases, the female to male ratio was 6.6:1 [3]. The diagnosis of AA is fairly difficult due to its nonspecific clinical and radiological findings [2]. Locally in Saudi Arabia, we have found only one study reported by Amr and El‐Mallah in 1995 regarding AA of the vagina with extensions into the perineum [4]. In our study, we report a case of aggressive angiomyxoma of the posterior wall of the uterus.

## Case presentation

**Figure 1:**
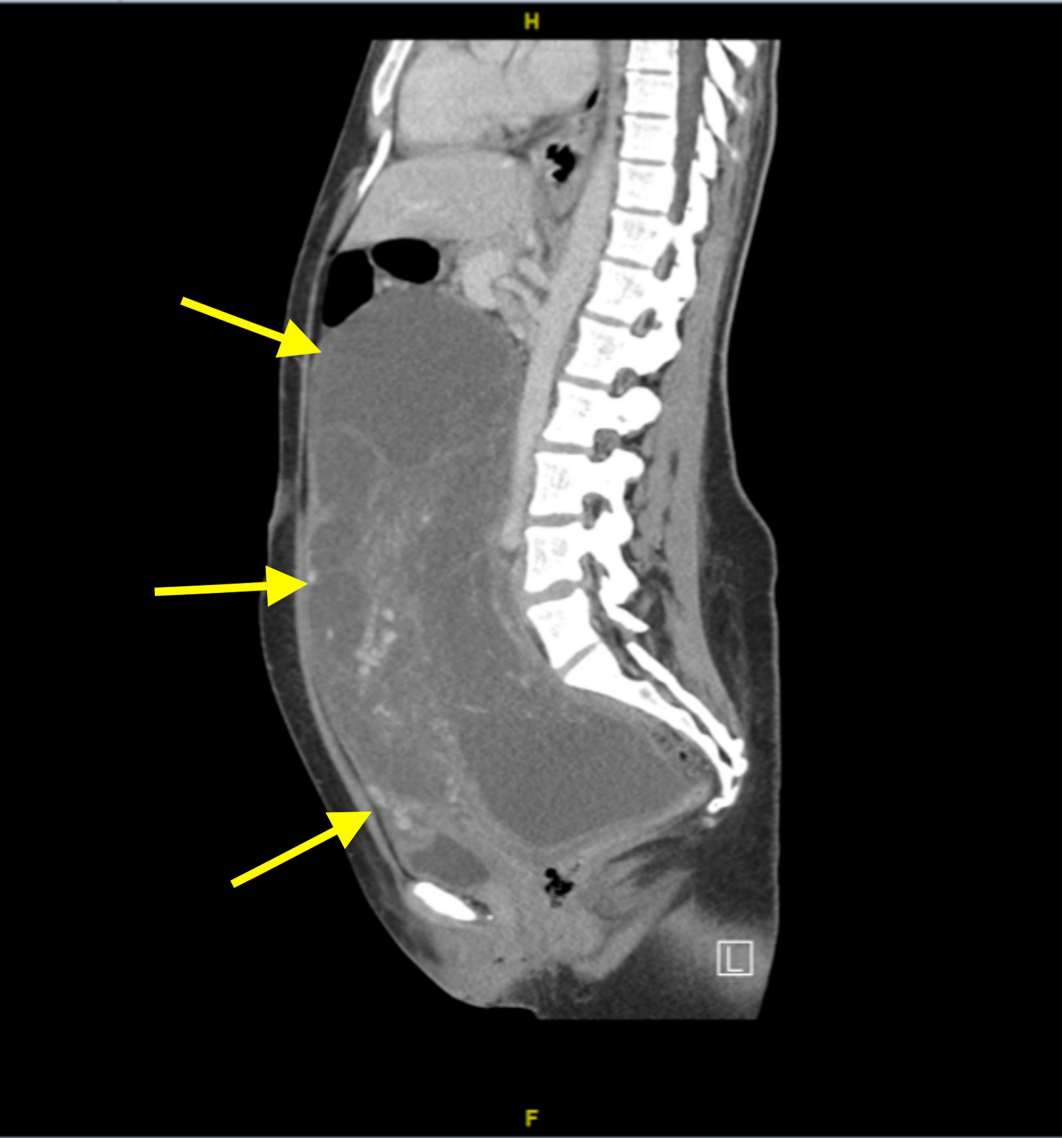
CT sagittal view of the abdomen and pelvis Showing the large mass extending from the pelvis to the abdomen.

**Figure 2:**
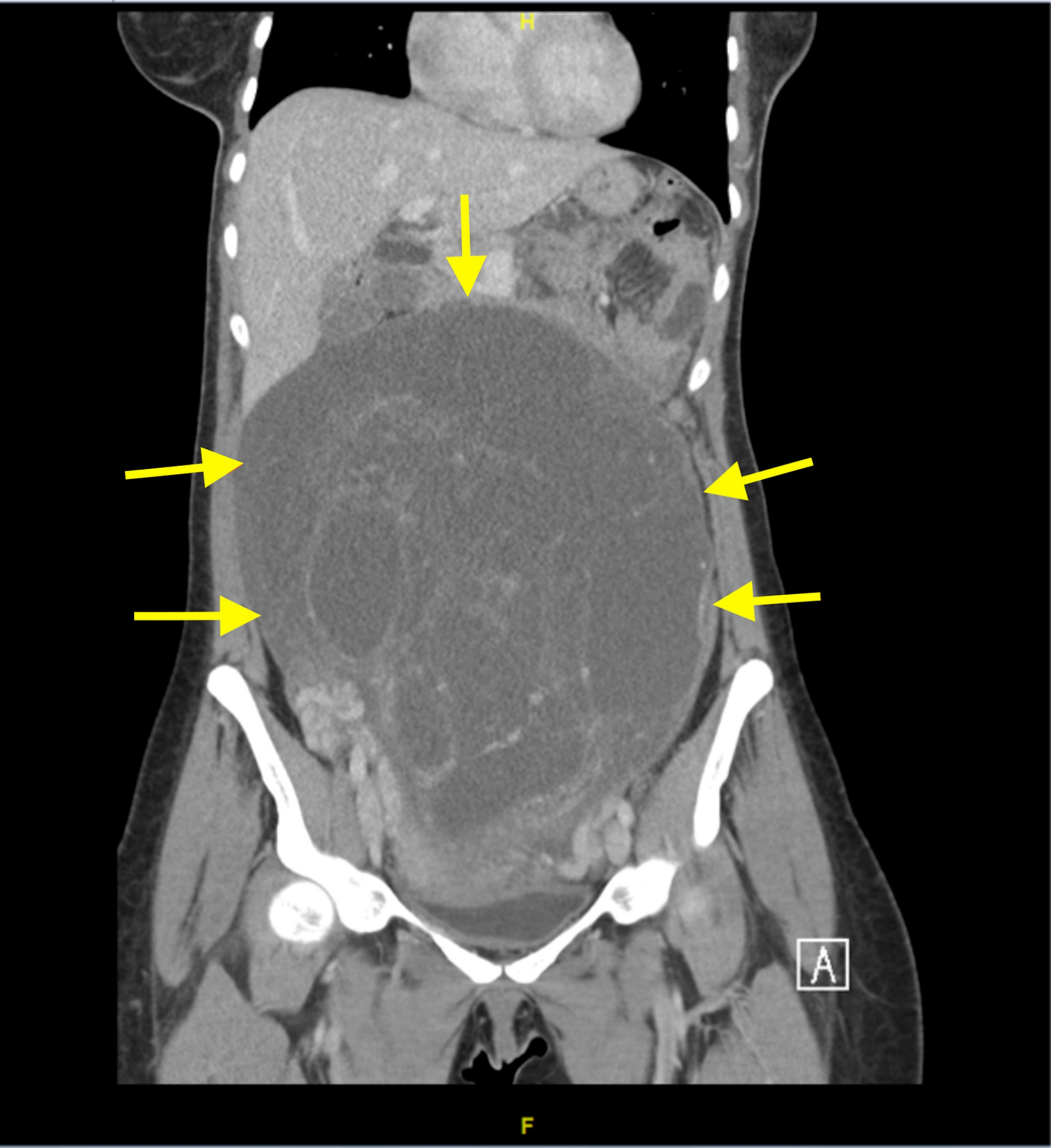
CT coronal view of the abdomen and pelvis Showing the large multiloculated abdominopelvic lesion.

The proband is a 35-year-old female patient G0P0, who was initially presented with a four-month history of abdominal pain and distention. She started to have abdominal pain back in February 2018. The abdominal pain was in the umbilical region. It was crampy and paroxysmal in nature with radiation toward the lower back, the paraumbilical region, and sometimes to the suprapubic region. Due to the vague presentation, she has been misdiagnosed with irritable bowel syndrome (IBS). A few months later the symptoms increased, and the pain increased four to six days prior to the menstrual period and was relieved during menstruation. The severity of the pain was nine out of ten, and it was not relieved by analgesics. The abdominal pain was associated with distention, severe heartburns, nausea, flatus, and change in her bowel habits. She had no bleeding nor any vaginal discharge. However, the abdominal distention was most noticeable, especially as it increased at a fast rate. She had a positive history of weight loss but no fever and no other constitutional symptoms. She also had a previous history of lower limb swelling on and off which was not present at the time of the diagnosis. Other systemic reviews were unremarkable. She had a past medical history of the diffusely enlarged thyroid gland. Past surgical history was unremarkable. She had a family history of breast cancer in her aunt, her mother had a thyroidectomy. Clinically, there was a palpable abdominal mass size 7 x 7 cm and fixed, with no tenderness. Inguinal lymph nodes were not palpable. There was no evident lower limb edema. Her routine hematological and biochemical screen was unremarkable. Tumor markers showed normal levels of alpha-fetoprotein (< 2.0 ng/mL), cancer antigen (CA) 15-3 (22.2 U/mL), CA 19.9 (16.0 U/mL), however, the level of CA 125 was high (50.3 U/mL). Beta human chorionic gonadotropin was negative (<12 mIU/mL). A computed tomography scan was performed in an outside center which demonstrated a huge abdominopelvic mass extending from the pelvis towards the upper abdomen. Multiple variable cystic lesions were also noted. Pelvic CT was done at our center, it showed a large multiloculated abdominopelvic lesion measuring 25.3 x 20 x 8.6 with multiple enhancing septa. No fat, soft tissue component, nor classifications were noticed (Figures 1, 2). 

**Figure 3:**
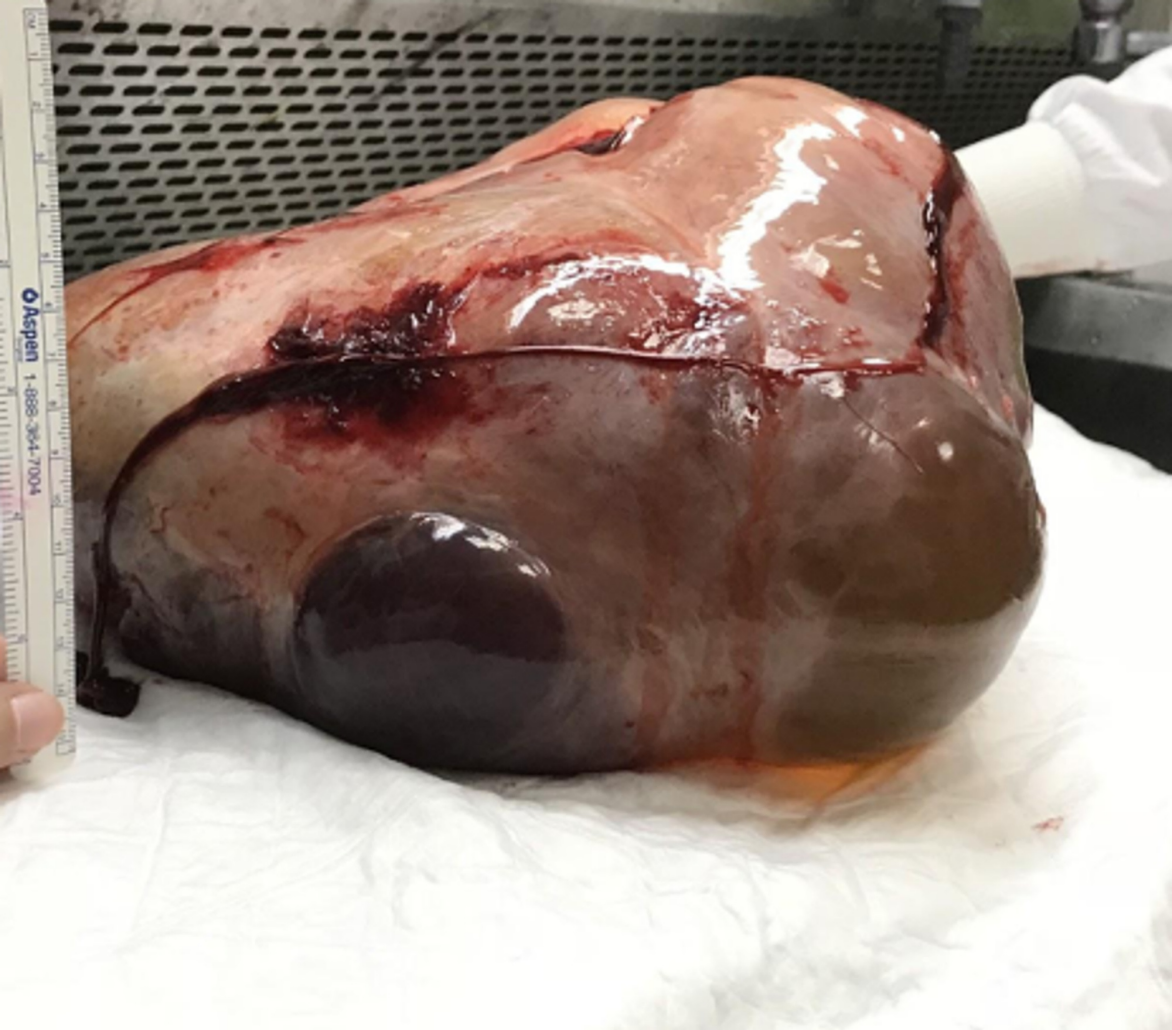
Mass of posterior wall of the uterus post-resection Showing solid myxoid gelatinous surface.

**Figure 4:**
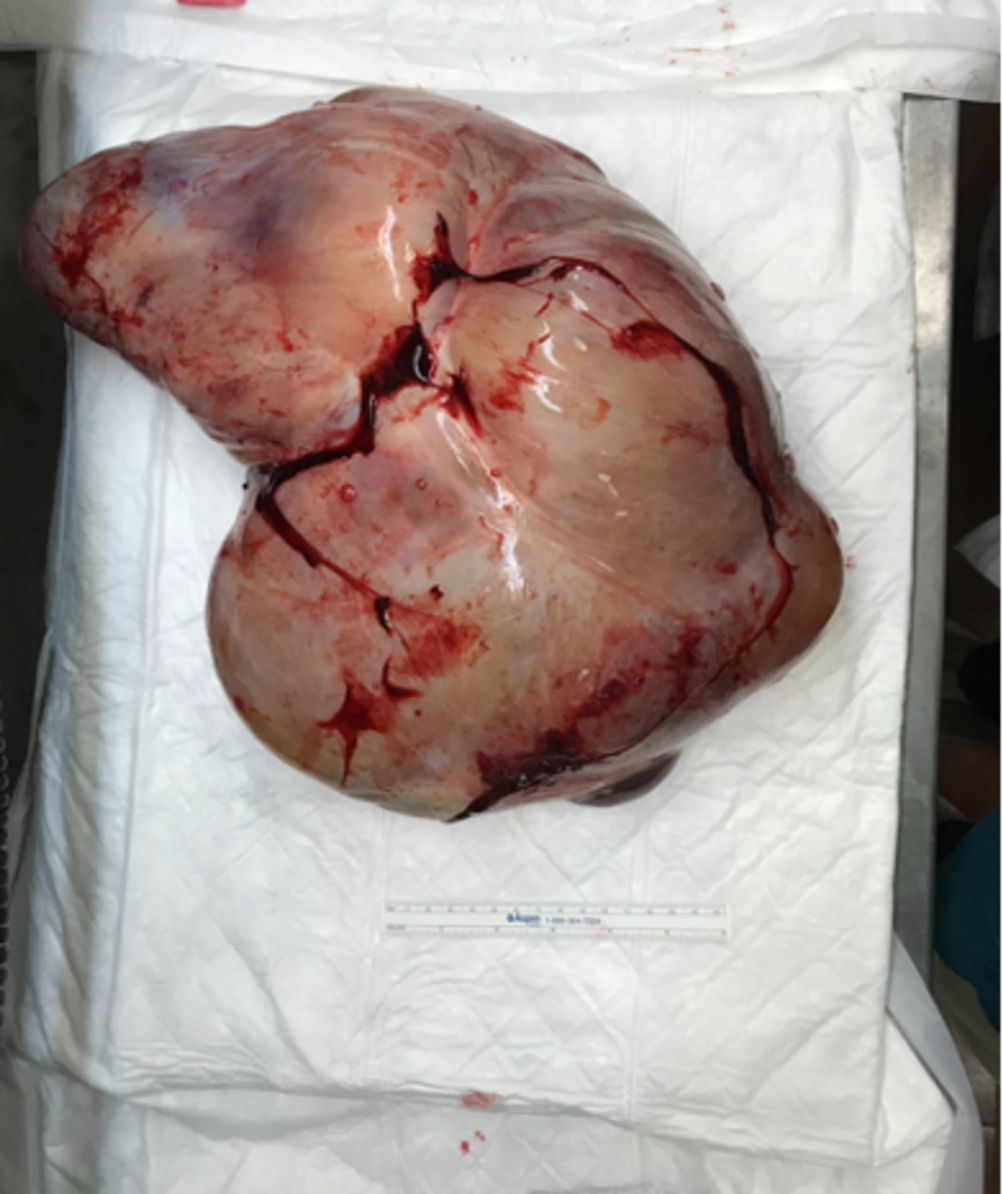
Mass of posterior wall of uterus post-resection Large mass with outer smooth surface measures 40 × 28 × 15 cm.

A chest CT was also performed, it showed no evidence of metastasis in the chest. The case was discussed in our institutional tumor board meeting and a laparotomy with possible hysterectomy was recommended. The findings were reported to the patient and her family, and consent was taken for surgery. The laparotomy was performed in July 2018, and posterior uterine mass resection revealed a large mass sized 40 x 28 x 15 cm with an outer smooth surface. The cut section showed a solid myxoid gelatinous surface with cystic areas (Figures 3, 4). 

Immunohistochemical staining was positive for caldesmon, estrogen receptors (ER+), progesterone receptors (PR+), desmin, smooth muscle actin, proliferative index Ki-67 8%, muscle-specific actin, vimentin, and was negative for S100 (a marker for Schwann cells, chondrocytes, adipocytes, and melanocytes cells). The diagnosis was confirmed to be aggressive angiomyxoma and managed by mass resection with adjuvant hormonal treatment (raloxifene).

One year after surgical excision and hormonal therapy, the patient presented with recurrent abdominal pain. A multiaxial enhanced CT scan of the chest, abdomen, and pelvis in the porto-venous phase was performed and a comparison was made with previous results to rule out recurrence. Chest CT revealed an enlarged heterogeneous thyroid. Other than that, there was no mediastinal or axillary lymphadenopathy. No pleural or pericardial effusion and the lung parenchyma showed no suspicious nodules or masses. Moreover, the major vessels were not compressed by the mass. However, the CT abdomen demonstrated a well-defined hypodense ring-enhancing lesion in the posterior pelvis at the site of previously placed surgical clips. It measured 3.4 x 3.4 x 3 cm in its anteroposterior, transverse, and craniocaudal dimensions. Minimal pelvic free fluid and no suspicious bony lesions were noted. The possible suspected etiology of the lesion included a loculated collection, however, recurrence could not be ruled out. Therefore, clinical and tumor marker correlation was required. Findings of the liver, kidneys, and spleen were unremarkable. It was followed-up clinically and pelvic magnetic resonance imaging was performed. The MRI ruled out local recurrence and noted that the tubular-shaped fluid containing structure within the left adnexal was more suggestive of hydrosalpinx.

## Discussion

Aggressive angiomyxoma is a rare benign slow-growing mesenchymal tumor that tends to arise in the female vulvovaginal and perineal region, and analogous perineal and inguinoscrotal regions in males [1,5]. It was first reported by Steeper and Rosai in 1983, who described a case series of nine female patients each had an infiltrative benign-appearing myxoid and vascular tumor with a high tendency for local recurrence (up to 71%), hence the name aggressive [6]. The majority of cases are reproductive-aged women with a peak incidence between the third and fourth decades of life, which suggests they are at higher risk [7-10]. Extragenital involvement is rare with only few reported cases [11]. The clinical manifestation of AA is nonspecific, thus, it is sometimes mistaken with entities, such as Bartholin cyst, lipoma, hernia, or like in our case she was misdiagnosed as IBS [6]. 

On imaging, AA is a smooth well-defined soft tissue tumor that tends to displace surrounding organs without infiltrating them [12]. On ultrasonography, AA appears as a cystic or hypoechogenic lesion [13]. Computed tomography scan is non-specific and shows variable appearances of AA, it might appear as a hypodense lesion, as a well-defined homogeneous mass, or as a predominant cystic lesion with solid components [12,14]. AA shows significant contrast enhancement on magnetic resonance imaging (MRI), which is due to its high vascularity [12,14]. In addition, the tumor appears as an isointense or hypointense lesion on T1-weighted images, and hyperintense on T2-weighted images; owing to its high water content and loose myxoid matrix [12,14].

Histologically, AA appears as a hypocellular highly vascular tumor within a myxoid stroma that contains stellate or spindled cells [1,2]. The presence of positive estrogen and progesterone receptors in patients’ tumor cells suggests a hormonal involvement in the development of the tumor [7,8]. However, a variety of vulvar lesions can also be positive for these receptors, suggesting that ER or PR immunoreactivity cannot be used to distinguish aggressive angiomyxoma and its histological mimics [15]. Immunohistochemically, AA has no specific markers [16]. However, there is general immunopositivity for vimentin and desmin [16]. S100 protein is not a feature of AA but may be present [16]. On the other hand, tumor cells of male patients described in literature did not express estrogen and progesterone receptors but were immunoreactive for desmin, smooth muscle actin, and vimentin [17]. Finally, chromosomal translocation of the long arm (q) of chromosome 12 at position 13-15 (12q13-15) band involving the high mobility group AT-hook 2 (HMGA2) gene has been described in relation to AA [2]. 

AA has different therapeutic approaches [16]. Currently, there is no global consensus on the preferred management of AA and complete surgical excision remains the treatment of choice [16]. The main issue is the high rate of local recurrence following surgical excision, with an incidence rate of 36-72% [6,13,18]. The use of gonadotropin-releasing hormone agonists has been suggested as a neoadjuvant or as an adjuvant to prevent recurrence in these tumors, however, the effectiveness of this option is lacking [1,2]. Meanwhile, radiation therapy is a potential alternative to extensive resection in advanced disease or as an adjuvant for recurrent tumors, though its use as the only therapeutic approach remains controversial [7]. 

The morbidity and mortality of the AA depend mainly on its localization [19]. The majority of cases have a good prognosis with a very rare chance of metastases; not more than two cases have been reported in the literature [19]. Finally, long-term follow-up is recommended in all patients who underwent resection [20]. These follow-ups depend on clinical examinations, although MRI might be required under certain circumstances as a preferred method to detect early recurrence [20]. However, the frequency and modalities used in follow-ups have not been well established and more cases are needed [20].

## Conclusions

In conclusion, we describe a case of aggressive angiomyxoma of the posterior wall of the uterus in a Saudi female patient, which has a few reported cases and mentioned the clinical approach in our center. The current known treatment of choice is total surgical excision, however, other approaches such as radiation and hormonal therapy are emerging as potential future alternatives. Due to its high recurrence rate, long-term follow up is necessary and MRI might be indicated.
